# The prognostic value of four interleukin-1 gene polymorphisms in caucasian women with breast cancer – a multicenter study

**DOI:** 10.1186/1471-2407-9-78

**Published:** 2009-03-06

**Authors:** Christoph Grimm, Eva Kantelhardt, Georg Heinze, Stephan Polterauer, Robert Zeillinger, Heinz Kölbl, Alexander Reinthaller, Lukas Hefler

**Affiliations:** 1 Department of Obstetrics and Gynecology, Medical University of Vienna, Vienna, Austria; 2 Department of Obstetrics and Gynecology, Martin-Luther-University Halle-Wittenberg, Halle, Germany; 3 Core Unit for Medical Statistics and Informatics, Medical University of Vienna, Vienna, Austria; 4 Department of Obstetrics and Gynecology, Johannes Gutenberg University of Mainz, Mainz, Germany

## Abstract

**Background:**

The proinflammatory cytokine interleukin-1 (IL-1) is known to play an important role in the carcinogenesis of breast cancer. Although IL-1 gene polymorphisms were reported to be associated with increased risk of breast cancer, their influence on survival of Caucasian breast cancer patients remains to be shown.

**Methods:**

We studied the influence of four common gene polymorphisms (*IL1A *-889C/T, *IL1B *-511C/T, *IL1B *+3953E1/E2, and *IL1RN *long/2) of the IL-1 family on survival in 262 Caucasian patients with breast cancer by univariate and multivariate survival analysis. The combined effect of the four gene polymorphisms on overall survival was studied by haplotype analysis.

**Results:**

In the present study 38 cases of cancer related death and a median time of follow-up (range) of 55.3 (0.4–175.8) months was observed. *IL1RN *2/2 (homozygous mutant) gene polymorphism was associated with shortened disease free and overall survival in a univariate (p = 0.001 and p = 0.01, respectively) and multivariate analysis (p = 0.002, Odds Ratio [95% Confidence Interval] = 3.6 [1.6–8.0] and p = 0.05, Odds Ratio = 3.0 [1.1–9.3], respectively). Presence of the homozygous mutant genotype of the *IL1A *-889 and *IL1B *+3953 gene polymorphism was associated with overall survival in the univariate (p = 0.004 and p = 0.002, respectively), but not in the multivariate analysis. No association was observed between all possible haplotype combinations and overall survival.

**Conclusion:**

Carriage of the mutant alleles of *IL1RN *was independently associated with shortened disease free and overall survival rates in Caucasian patients with breast cancer.

## Background

The interleukin-1 (IL-1) family consists of the cytokines, IL-1alpha (IL-1a), IL-1beta (IL-1b) and a specific receptor antagonist (IL-1RA) [1]. IL-1a and IL-1b are involved in various physiological and patho-physiological processes such as modulating the host response to microbial invasion, inflammation, and tissue injury [2]. IL-1RA neutralizes IL-1 action by binding to the IL-1 receptors (IL-1RI and IL-1RII) [3].

In addition to its role in inflammatory processes, IL-1 has been extensively studied in various malignancies. Expression of IL-1 has been described in breast cancer tissue [4,5] as well as several other tumor tissues [6,7]. Its production and release by tumor cells results in an autocrine and paracrine induction of prometastatic genes in human breast cancer [8]. *In vitro *studies reported IL-1 to be crucially involved in cell survival, proliferation, and angiogenesis [6]. Recent studies suggested that IL-1 was associated with more aggressive forms of breast cancer [9,10].

The *IL-1 *gene cluster on chromosome 2q14.2 comprises 3 related genes within a 430-kilobase (kb) region, *IL-1A*, *IL-1B*, and *IL-1RN*, which encode the pro-inflammatory cytokines IL-1a, IL-1b, and their endogenous receptor antagonist IL-1RA, respectively [11]. *In vitro *and *in vivo *studies demonstrated that *IL1-A *and *IL1-B *gene polymorphisms [12,13] corresponded with altered IL-1a and IL-1b protein expression, respectively [12,14]. Regarding the IL1 receptor antagonist, the *IL1RN *mutant allele 2 has been repeatedly associated with changes of the IL-1RA protein expression [15,16].

*IL-1 *polymorphisms have been investigated in a variety of malignancies. They were associated with an increased risk for ovarian [17], gastric [18], lung [19], and prostate cancer [20]. With respect to breast cancer, two recent studies demonstrated an association between *IL-1 *polymorphisms and increased risk for the disease [21,22].

While other interleukin polymorphisms have been extensively studied as prognostic markers in breast cancer patients [23-25] little is known about the prognostic value of *IL-1 *gene polymorphisms. In one study comprising Tunisian patients with breast cancer the *IL1A *TT (homozygous mutant) genotype was associated with impaired prognosis in patients with breast cancer [26].

To the best of our knowledge, we are the first to investigate the prognostic value of gene polymorphisms within the *IL-1 *family in Caucasian patients with breast cancer.

## Methods

Two-hundred-sixty-two consecutive patients with breast cancer treated between 1999 and 2001 at the Departments of Obstetrics and Gynecology, Martin-Luther-University Medical School, Halle-Wittenberg, Germany and Medical University of Vienna, Vienna, Austria, were included in our study. All of the participating patients were of Caucasian origin and signed written consent. Approval for this study was obtained by the Institutional Review Boards at the Martin-Luther-University Medical School, Halle-Wittenberg, Germany and the Medical University of Vienna, Vienna, Austria.

Patients' data was collected by reviewing patients' files. Of note, family history of breast cancer was documented only in very few cases and therefore not included in our analysis. Histological staging of breast cancer was performed according to the current classification of the International Union Against Cancer (UICC). All cases were reviewed by an experienced pathologist, blinded to patients' clinical data. Patients received surgery, chemotherapy, radiotherapy, and endocrine therapy by standard protocols. National guidelines were followed according to St. Gallen recommendations in the respective years.

DNA was extracted from patients' blood. Pyrosequencing and polymerase chain reaction (PCR) were performed according to established protocols as described previously [27].

After testing for normality using Kolmogorov-Smirnov test, values are given as mean (standard deviation [SD]) or median (range), where appropriate. Hardy-Weinberg equilibrium was tested by chi-square tests comparing observed and expected haplotype frequencies. Associations between polymorphisms and clinicopathological parameters were calculated by chi-square tests – results are given as p-value and Odds Ratio (OR) (95% Confidence Interval [95%CI]). Due to multiple testing Bonferroni-Holmes adjustment was performed. Parameters have been calculated as followed: *IL1A *-889 (C/C and C/T vs. T/T), *IL1B *-511 (C/C and C/T vs. T/T), *IL1B *+3953 (E1/E1 and E1/E2 vs. E2/E2), *IL1RN *VNTR intron 2 (long/long and long/2 vs. 2/2), tumor status (pT1 vs. pT2-4), tumor grade (well differentiated vs. moderately and poorly differentiated), lymph node involvement (yes vs. no), presence of estrogen or progesterone receptor (positive vs. negative), and patient's age at diagnosis (≤ 50 years vs. > 50 years).

Survival probabilities were calculated by the product limit method of Kaplan and Meier. Differences between groups were tested using the log-rank test. The results were analyzed for the endpoint of disease free and overall survival. Survival times of patients without any evidence of recurrent disease or still alive at the time of last follow-up were censored with the last follow-up date. A multivariate Cox regression model was performed for disease free and overall survival comprising tumor status, tumor grade, lymph node involvement, presence of estrogen or progesterone receptor, and in the respective univariate analysis significantly associated gene polymorphisms (i.e., *IL1RN *VNTR intron 2 gene polymorphism in the disease free survival analysis and *IL1A *-889C/T, *IL1B *+3953, and *IL1RN *VNTR intron 2 gene polymorphisms in the overall survival analysis).

Haplotype frequencies were calculated by using SAS/Genetics software. Association between haplotypes and breast cancer survival was calculated using a Cox regression model treating each haplotype as an independent continuous variable.

We used the software SAS System (Version 9.1 SAS Institute Inc., Cary, NC) and SPSS (SPSS 11.0, SPSS Inc. Chicago, IL) for statistical analysis. Two-sided p-values ≤ 0.05 were considered statistically significant.

## Results

Patients' characteristics are given in Table 1 (Additional file 1). Genotype and allele frequencies of the four investigated *IL-1 *gene polymorphisms are given in Table 2 (Additional file 1). As previously published genotype distribution of all investigated gene polymorphisms was in Hardy-Weinberg equilibrium [28]. The four investigated polymorphisms were all in linkage disequilibrium (*IL1A *-889 and *IL1B *-511: p = 0.0001, Lewontin's D' = 0.5; *IL1B *-511 and *IL1B *+3953: p < 0.0001, Lewontin's D' = 0.7; *IL1B *+3953 and *IL1RN*: p = 0.004, Lewontin's D' = 0.6).

*IL1A *-889, *IL1B *-511, *IL1B *+3953, and *IL1RN *were not associated with clinicopathological parameters, i.e., tumor status (p = 0.5, p = 0.8, p = 0.2, p = 0.3), tumor grade (p = 0.9, p = 0.9, p = 0.3, p = 0.3), lymph node involvement (p = 0.6, p = 0.8, p = 0.8, p = 0.9), presence of estrogen or progesterone receptor (p = 0.9, p = 0.7, p = 0.9, p = 0.9), and patient's age at diagnosis (p = 0.2, p = 0.4, p = 0.7, p = 0.8), respectively.

**Figure 1:**
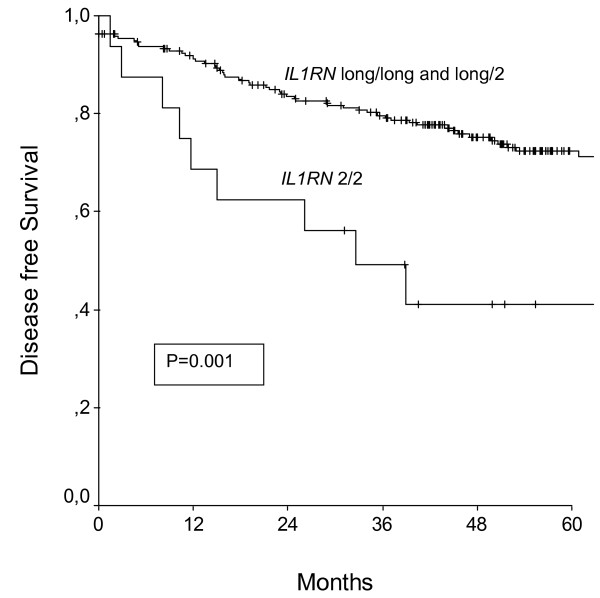
**Disease free survival of patients with breast cancer broken down by the *IL1RN *polymorphism**. P denotes the log-rank test value.

**Figure 2:**
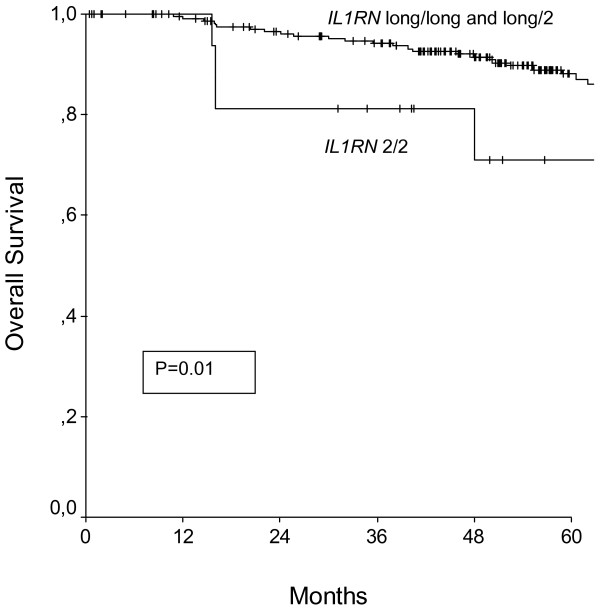
**Overall survival of patients with breast cancer broken down by the *IL1RN *polymorphism**. P denotes the log-rank test value.

Survival analysis were calculated by performing univariate Kaplan-Meier analysis and multivariate Cox-Regression models to correct for established clinicopathological parameters. Results are presented in Table 3 (Additional file 1). In a univariate Kaplan-Meier analysis only *IL1RN *gene polymorphisms was associated with disease free survival (Figure 1.), whereas *IL1A *-889, *IL1B *-+3953, and *IL1RN *(Figure 2.) were associated with overall survival. After correcting for established clinicopathological parameters in a multivariate Cox-Regression model, the *IL1RN *gene polymorphism was independently associated with shortened disease free and overall survival.

Furthermore, the combined effect of the four gene polymorphisms on breast cancer survival was investigated by haplotype analysis (Additional file – Table 4). No significant associations between haplotypes and overall survival were observed.

## Discussion

In the present study, the *IL1RN *long/2 gene polymorphism was independently associated with an unfavourable prognosis in breast cancer patients, while presence of the mutant alleles of *IL1A *-889 and *IL1B *+3953 were only associated with a shortened overall survival in a univariate analysis. These findings are in line with a series of studies that have found interleukin gene polymorphisms to be associated with shortened overall survival in breast cancer patients [23,25].

This is biologically plausible, as mutations of *IL1A *-889 and *IL1B *+3953 are thought to lead to increased IL-1 production [12,14,15]. In comparison, the mutant *IL1RN *allele has been reported to modify the binding on the IL1-receptor, leading to a worse inhibition of the IL-1a and IL-1b binding and subsequently to an increased IL-1 production [29]. This might result in an unfavourable proinflammatory status and enhanced tumor aggressiveness, which is likely to result in a shortened survival.

In addition to its potential as a prognostic marker, particularly *IL1RN *is of increasing interest with regard to therapeutic implications. Recently a recombinant human IL-1 receptor antagonist, has been approved to block the proinflammatory effects of IL-1 in patients with rheumathoid arthritis [30]. Experimental data based on mouse models support the hypothesis that an IL-1 receptor antagonist might also work as a therapeutic agent in neoplasms [30,31].

Of note, the exact mechanisms by which these four polymorphisms exert their effects on IL-1 levels remain still unclear to date. In the present investigation, we focused on the evaluation of *IL-1 *gene polymorphisms, and did not plan to investigate serum levels of the IL-1 cytokines. Interestingly, *IL1RN *was the only of three polymorphisms that remained significant in the multivariate model, which was performed to correct for potential confounders. Despite this consistent association between *IL1RN *and overall survival in a univariate and multivariate analysis, it has to be considered that seven variables were included in the multivariate analysis with only 38 events observed. The high number of variables was included in the multivariate model as this is the only way to evaluate an independent association between the respective gene polymorphism and overall survival. In that case, however, a random finding can not be completely ruled out. The other two polymorphisms, *IL1A *-889 and *IL1B *+3953, were only associated with shortened survival in a univariate survival analysis. Therefore, it can not be ruled out that these associations might result from unequal distribution of well known prognostic factors, such as tumor stage, lymph node involvement or tumor grade.

Of note, it was not feasible to evaluate the association between *IL-1 *gene polymorphisms and family history of breast cancer as this information was missing in the majority of patients. Furthermore, we were unable to investigate the association between *IL-1 *gene polymorphisms and distant metastases because of the limited number of events (4 patients with distant metastases).

As all four *IL-1 *gene polymorphisms were in linkage disequilibrium, we performed a haplotype survival analysis to investigate whether there is a high risk haplotype combination. Interestingly, we were not able to identify such a high risk haplotype combination, which might be caused by the relatively high number of variables (n = 16) compared to the number of events (n = 38 cancer related deaths).

## Conclusion

IL-1 has been reported to be crucially involved in cell survival, proliferation, and angiogenesis in cancer cells [6]. Moreover IL-1 secretion seems to be associated with a more aggressive form of breast cancer [9,10]. *IL-1 *gene polymorphisms were reported to be associated with an increased risk of breast cancer [21,22]. They are thought to increase IL-1 levels subsequently contributing to a proinflammatory environment. All of the four investigated *IL-1 *gene polymorphisms have been demonstrated to correspond with altered IL-1 protein expression [12-16]. The *IL1RN *gene polymorphism might be of prognostic value as carriage of the two mutant alleles was independently associated with shortened disease free and overall survival in Caucasian patients with breast cancer.

## Competing interests

The authors declare that they have no competing interests.

## Authors' contributions

CG was critically involved in designing the study, statistical analysis, and drafted the manuscript. LH conceived of the study, participated in the design of the study and helped draft the manuscript. EK carried out molecular genetic studies, contributed to acquisition of data, and critically revised the manuscript. GH was involved in design of the study, statistical analysis, and revision of the methods section of the manuscript. SP was involved in acquisition of the data, and interpretation of the data. RZ participated in primer selection, provided biomolecular expertise, carried out molecular analysis, and helped with statistical analysis. HK partially conceived of the study and participated in its design and coordination. AR was substantially involved in the design of the study and drafting the manuscript. All authors read and approved to the final manuscript.

## Pre-publication history

The pre-publication history for this paper can be accessed here:

http://www.biomedcentral.com/1471-2407/9/78/prepub

## Supplementary Material

Additional file 1 **Tables**. Table 1. Characteristics in patients with breast cancer. Table 2. Genotype and allele frequencies of the four investigated *IL1 *gene polymorphisms in breast cancer patients. Table 3. Survival analysis of *IL1 *gene polymorphisms and prognostic covariates in patients with breast cancer. Table 4. Association between interleukin-1 haplotypes and overall survival of patients with breast cancer. Click here for file
