# Supplementary material for: The prognostic value of four interleukin-1 gene polymorphisms in caucasian women with breast cancer – a multicenter study
Source: BMC Cancer. 2009 Mar 6;9:78. doi: 10.1186/1471-2407-9-78 (PMC2656545; doi:10.1186/1471-2407-9-78)
Supplement: Additional file 1 — Tables. Table 1. Characteristics in patients with breast cancer. Table 2. Genotype and allele frequencies of the four investigated IL1 gene polymorphisms in breast cancer patients. Table 3. Survival analysis of IL1 gene polymorphisms and prognostic covariates in patients with breast cancer. Table 4. Association between interleukin-1 haplotypes and overall survival of patients with breast cancer. [file 1471-2407-9-78-S1.doc]

**Table1.** Characteristics in patients with breast cancer.

| **Characteristic** | **No. of patients** |
| --- | --- |
|  |  |
| **Total number of patients enrolled** | 262 |
| **Age at diagnosis (years)** | 55.9 (12.7)* |
| **Tumor status** |  |
| pT1 | 131 |
| pT2 | 98 |
| pT3 | 11 |
| pT4 | 19 |
| Unknown | 3 |
| **Tumor grade** |  |
| Well differentiated | 19 |
| Moderately differentiated | 145 |
| Poorly differentiated | 96 |
| Unknown | 2 |
| **Lymph node involvemen**t |  |
| No | 150 |
| Yes | 110 |
| Unknown | 2 |
| **Histological type** |  |
| Ductal carcinoma | 161 |
| Lobular carcinoma | 36 |
| Tubular carcinoma | 7 |
| Medullary carcinoma | 6 |
| Mucinous carcinoma | 5 |
| Undifferentiated carcinoma | 9 |
| Papillotubular carcinoma | 6 |
| Other | 32 |
| **Hormone receptor expression** |  |
| Estrogen receptor | 159 |
| Progesterone receptor | 140 |
| Estrogen and progesterone receptor | 171 |
| **Menopausal status** |  |
| Premenopausal | 96 |
| Postmenopausal | 163 |
| Unknown | 3 |
| **No. of patients with follow-up information available** | 259 |
| Dead as result of disease (=events) | 38 |
| Dead as result of other causes | 2 |
| Alive with no evidence of disease or stable disease | 219 |
| Median time of follow-up (months) | 55.3 (0.4-175.8)** |
| **Recurrence status** |  |
| No. of patients with recurrent disease | 73 |
| Mean time to recurrent disease (months) | 43.3 (25.6)* |

*Mean (Standard Deviation)

**Median (Range)

**Table 2.** Genotype and allele frequencies of the four investigated *IL1* gene polymorphisms in breast cancer patients.

| ***IL1* gene polymorphisms** | **No. of patients (%)** | **p-value (HWE*)** |
| --- | --- | --- |
|  |  |  |
| ***IL1A* -889** |  |  |
| C/C | 120 (45.8%) | 0.2 |
| C/T | 120 (45.8%) |  |
| T/T | 22 (8.4%) |  |
| Alleles |  |  |
| C | 360 (68.7%) |  |
| T | 164 (31.3%) |  |
| ***IL1B* promoter -511** |  |  |
| C/C | 121 (46.2%) | 0.5 |
| C/T | 110 (42.0%) |  |
| T/T | 31 (11.8%) |  |
| Alleles |  |  |
| C | 352 (67.2%) |  |
| T | 172 (32.8%) |  |
| ***IL1B* exon 5 position +3953** |  |  |
| E1/E1 | 156 (59.5%) | 0.7 |
| E1/E2 | 93 (35.5%) |  |
| E2/E2 | 13 (5.0%) |  |
| Alleles |  |  |
| E1 | 405 (77.3%) |  |
| E2 | 119 (22.7%) |  |
| ***IL1RN* VNTR intron2** |  |  |
| long/long | 151 (57.6%) | 0.8 |
| long/2 | 95 (36.3%) |  |
| 2/2 | 16 (6.1%) |  |
| Alleles |  |  |
| 240 base pairs (bp) | 127 (24.2%) |  |
| 325 bp | 1 (0.001%) |  |
| 410 bp | 385 (73.5%) |  |
| 500 bp | 11 (2.1%) |  |

*HWE= Hardy-Weinberg equilibrium

**Table 3.** Survival analysis of *IL1* gene polymorphisms and prognostic covariates in patients with breast cancer.

|  | **Disease free survival** | | | |  | **Overall survival** | | | |
| --- | --- | --- | --- | --- | --- | --- | --- | --- | --- |
|  | **Univariate** |  | **Multivariate** | |  | **Univariate** |  | **Multivariate** | |
|  | **P** |  | **P** | **HR (95% CI)*** |  | **P** |  | **P** | **HR (95% CI)*** |
| Tumor status(pT1 vs. pT2-4) | 0.0003 |  | 0.3 | 1.4 (0.8-2.5) |  | <0.0001 |  | 0.03 | 3.8 (1.1-13.4) |
| Tumor grade(G1 vs. G2 and G3) | 0.09 |  | 0.5 | 1.6 (0.4-7.4) |  | 0.08 |  | 0.9 | 264724.8 (0.0-∞) |
| Estrogen or progesterone receptor(negative vs. positive) | 0.04 |  | 0.02 | 0.5 (0.3-0.9) |  | 0.006 |  | 0.002 | 0.3 (0.1-0.7) |
| Lymph node involvement(negative vs. positive) | <0.0001 |  | <0.0001 | 2.8 (1.6-4.8) |  | 0.002 |  | 0.05 | 2.5 (1.2-6.0) |
| *IL1A* -889 C/T (C/C and C/T vs. T/T) | 0.1 |  | -*** | - |  | 0.004** |  | 0.9 | 1.1 (0.2-7.9) |
| *IL1B* promoter -511 C/T (C/C and C/T vs. T/T) | 0.7 |  | -*** | - |  | 0.9 |  | -*** | - |
| *IL1B* exon 5 position +3953 (E1/E1 and E1/E2 vs. E2/E2) | 0.3 |  | -*** | - |  | 0.002** |  | 0.2 | 4.7 (0.6-40.1) |
| *IL1RN* VNTR intron2 (long/long and long/2 vs. 2/2) | 0.001** |  | 0.002** | 3.6 (1.6-8.0) |  | 0.01** |  | 0.05** | 3.0 (1.1-9.3) |

* Hazard Ratio (95% Confidence Interval)

** mutant allele associated with shortened survival

*** not included in the multivariate Cox-Regression model

**Table 4.** Association between interleukin-1 haplotypes and overall survival of patients with breast cancer.

| **Haplotypes** | **Haplotype frequency** | **Overall survival** | |
| --- | --- | --- | --- |
| *(IL1A* -889, *IL1B* -511, *IL1B* +3953, *IL1RN)* |  | *P* | HR (95% CI)* |
| mut-mut-mut-mut | 0.3% | 0.9 | 0.0 (-) |
| mut-mut-mut-wt | 2.0% | 0.5 | 53.9 (0.0-1.1^7^) |
| mut-mut-wt-mut | 1.2% | 0.7 | 0.6 (0.08-4.7) |
| mut-mut-wt-wt | 2.5% | 0.5 | 0.4 (0.03-5.7) |
| mut-wt-mut-mut | 2.2% | 0.4 | 2.8 (0.3-28.0) |
| mut-wt-mut-wt | 15.9% | 0.9 | 1.1 (0.4-2.8) |
| mut-wt-wt-mut | 2.1% | 0.3 | 0.2 (0.01-4.4) |
| mut-wt-wt-wt | 5.1% | 0.3 | 2.8 (0.5-17.4) |
| wt-mut-mut-mut | 0.0% | - | - |
| wt-mut-mut-wt | 0.4% | 0.8 | 0.03 (0.0-6.0^12^) |
| wt-mut-wt-mut | 13.2% | 0.3 | 1.8 (0.6-5.2) |
| wt-mut-wt-wt | 13.3% | 0.6 | 0.7 (0.1-3.3) |
| wt-wt-mut-mut | 0.1% | 0.06 | - |
| wt-wt-mut-wt | 1.9% | 0.06 | 0.0 (0.0-3449.2) |
| wt-wt-wt-mut | 5.2% | 0.2 | 0.03 (0.0-10.7) |
| wt-wt-wt-wt** | 34.6% | - | 1 (reference) |

*Hazard Ratios (95% Confidence Interval) were calculated in a Cox regression model by treating the frequency of each haplotype as a continuous independent variable.

** The “wt-wt-wt-wt” haplotype was used as the baseline haplotype.

wt=wild type, mut=mutant
